# Role of the 820 A/G variant in the *IGF-2* gene and recurrent spontaneous abortion in southern Iran: A cross-sectional study

**DOI:** 10.18502/ijrm.v13i9.7669

**Published:** 2020-09-20

**Authors:** Farzaneh Ardeshir, Leila Keshavarz, Fatemeh Asadian, Gohar Omidmokhtarkhanloo, Majid Yavarian

**Affiliations:** ^1^Department of Biology, Islamic Azad University, Arsanjan Branch, Arsanjan, Iran.; ^2^Department of Pathology, Shiraz University of Medical Sciences, Shiraz, Iran.; ^3^Persian Bayan Gene Research and Training Center, Dr. Faghihi’s Medical Genetic Center, Siraz, Iran.; ^4^Shiraz Nephron-Urology Research Center, Shiraz University of Medical Sciences, Shiraz, Iran.

**Keywords:** Variant, IGF-2, Spontaneous abortion, Genomic imprinting, Gene expression

## Abstract

**Background:**

Insulin-like growth factor-2 (*IGF-2*) is a polypeptide growth factor and one of the first genes expressed prior to the implantation of the embryo, with its highest expression in the placental cells. Its activity strongly depends on the genomic imprinting, and the result of the loss of genetic imprinting is the termination of the early stages of embryonic development, which can lead to recurrent spontaneous abortion.

**Objective:**

This cross-sectional study aimed to investigate the role of 820A/G variant of the *IGF-2* gene and the probability to recurrent spontaneous abortion** (**RSA) in southern Iran.

**Materials and Methods:**

In this study, 50 aborted fetuses tissue for the study group and blood samples umbilical-cord from newborns as control group (n = 50) were collected from Shiraz-Iran (2017). The genotyping of the target point in the *IGF-2 *gene was performed by Real-time Polymerase Chain Reaction and analyzed through high-resolution melting (HRM) curve.

**Results:**

Based on the collected data (AA genotype = reference), allele “A” frequency in aborted fetus was 51% and control 68% as well as allele G 49% and 32%, respectively. Moreover, 27 aborted embryos (54%) were heterozygous (A/G) (OR = 3.274, 95% CI = 1.015-10.561, p = 0.04), while 18 cases (36%) in control sample showed heterozygosity. Considering the phenotypic status, the G allele had a dominant effect on the incidence of RSA (p = 0.008, OR = 3.167).

**Conclusion:**

Based on the present study, the risk of abortion due to loss of heterozygosity or quantitative decline of the *IGF-2* is about three-fold in the southern Iran.

## 1. Introduction

Recurrent spontaneous abortion (RSA) is referred to three or more times of spontaneous fetal death prior to the 20th wk of pregnancy (1, 2). Several genetic and non-genetic factors may induce RSA, such as polycystic ovarian syndrome and quantitative and structural chromosomal abnormalities. It is generally accepted that the most common factors in the first trimester abortion is caused by chromosomal abnormalities (3, 4). It is believed that etiological causes of RSA usually are unclear in 30-50% of cases (5). As a result, some idiopathic abortions may be caused by genetic variant that changes in specific genes. These variations, so-called imprinted genes, could affect maternal or paternal genome activity and lead to RSA (6). Genomic imprinting is an epigenetic modification that affects gene expression in paternally or maternally originated genes. This process contradicts Mendelian inheritance. These genes do not have biallelic expression and only one allele is expressed in the genomic imprinting process, depending on the allelic origin of the parents (7).

In human, around 60 genes identified that is affected by the phenomenon of genetic imprinting (8, 9). Their expressions are critical during developmental fetal times. Many of them are regulatory genes involved in the growth and development of the fetus and are functionally hemizygous pattern. Therefore, alteration of effective copy number can cause developmental disorders.

Among these genes, the *Insulin-like growth factor-2* (*IGF-2*) gene at the position 11p15.5 is paternally expressed in fetus (10). The position in chromosome includes an imprinting region that contains the *IGF2* gene, which is consequently linked to the *H19* gene. The *IGF2*/*H19* expression is regulated by two imprinting control regions (ICR1 and ICR2). The ICR1 is a differentially methylated region at the upstream H19 promoter (11, 12). On the maternal allele, the CTCF protein, a zinc finger protein, binds to differentially methylated region (13, 14) and subsequently un-methylated maternal allele prevents *IGF-2* interaction with an *H19* enhancer at the downstream and inhibits *IGF-2* expression. Methylation of this region on a paternal allele changes the expression of gene profile and favors *IGF-2* expression (15).* IGF-2* gene-encoded protein is involved in prenatal growth and is highly active during embryonic development. The activity of the *IGF-2* gene depends on the copy that is inherited from the father (16). *IGF-2* gene regulates the growth rate of the placenta and the embryo through angiogenesis (17-20), nutrient transfer (21), and inhibition of apoptosis (22), and its dysfunction can lead to miscarriage (23, 24). The *IGF-2 *gene has single nucleotide polymorphism in its different regions that affects its expression quantities and function. One of such known single nucleotide polymorphism is 820A/G variant (25). This variant in the father reduces gene expression and leads to abortion (26). The diagnosis of paternal 820 A/G-genotypes of the *IGF-2* gene helps to predict the prospect of fetal loss due to gene imprinting.

This study was intended to inspect the frequency and potential effects of alleles in women with recurrent abortions to open a way to prevent this public burden.

## 2. Materials and Methods 

### Samples

During a period of six months (May-October 2017), for a cross-sectional study, 232 dead fetuses were collected from the labor wards of Hafez and Zeinabiea Hospitals, Shiraz-Iran. Ninety one of collected fetuses were from mothers who had experienced at least two or more consecutive miscarriages. Additionally, 22 cases were excluded due to abnormal sonography or maternal abnormal lab data. All fetuses with abnormal karyotype or Noninvasive prenatal testing (NIPT) (19 cases) were excluded from the study as well. Finally, 50 fetal tissue samples with normal karyotype were enrolled and included for molecular analysis. A match group was randomly selected from 50 mothers who had at least one delivery after 37 wk gestation as the control group and blood samples were taken from the neonates. Cases with non-spontaneous abortions as well as known disorders or anatomical abnormalities were excluded from the study. Demographic data of dead fetuses and controls presented in table I.

About 1 cm of aborted fetal tissue was stored at -20°C and used for DNA extraction. Samples were collected through questionnaire requiring information with spouse at the time of admission.

### Genetic analysis

Genomic DNA was extracted using PureLink genomic DNA kit (Life Technologies, CA, USA) according to the manufacturer's instruction. Quality and quantity of DNA were investigated using 0.8% agarose gel (Sigma-Aldrich, USA) electrophoresis and Nanodrop spectrophotometer device (BioTek Company,Germany). For the 820 A/G genotyping, Real-time Polymerase Chain Reaction was done with LC-Green (Idaho Technology, Salt Lake City, Utah, USA), and a high-resolution melting (HRM) analysis method following PCR amplification (Qiagen company, USA) was performed.

All samples were run along with positive and normal controls that were originally approved by Sanger sequencing method. Data were analyzed and genotyped using the HRM software.

### Real-time PCR reactions and melting analysis

**Figure 1:**
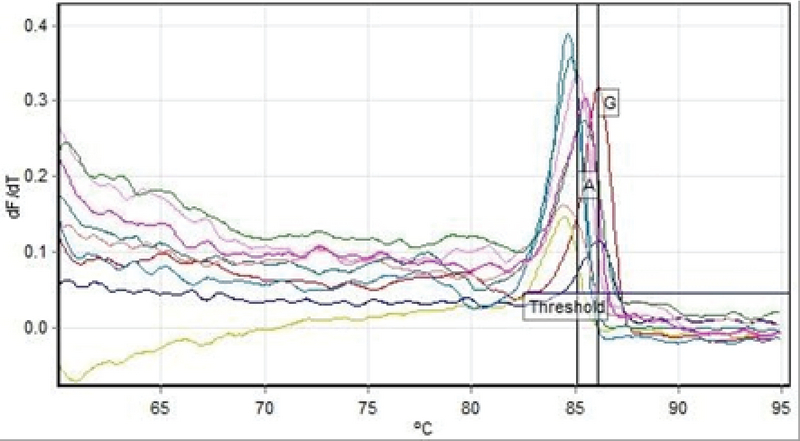
HRM analysis results to determine the genotype of the positional products polymorphisms 820 G/A in the *IGF-2* gene. The Real-time device shows diagram based on fluorescence variations relative to temperature variations. In this chart, each of the peaks represents the melting of a product PCR and, accordingly, determines the genotype.

A 10 µL reaction mixture with the Master Mix (2×) (Promega, Madison, WI, USA), LC Green Plus (10×) (Idaho Technology, Salt Lake City, Utah, USA) prepared. Final concentration of primers mixture and DNA was 10 µM and 200 pg/µL, respectively. The sequence of primers for *IGF-2 *(820A/G) design was done by online Primer 3 software. The sequence of designed primers are: (5'-CTT GAG TCC CTG AAC CAG CA-3') and (5'-TTC GGA TGG CCA GTT TAC CC-3'). The reaction PCR conditions were: initial cycle 95${}^{{}^{\circ}}$C for 90 sec, followed by 30 cycles of 95${}^{{}^{\circ}}$C for 25 sec, and annealing and extension temperature for 30 sec with final extension of 72${}^{{}^{\circ}}$C for 2 min. Melting analysis at the range of 65-95°C with an increment 0.1°C for 10 s was performed. Bio-Rad Precision Melt Analysis Software, version 1.2 (Bio-Rad Laboratories Inc., USA) was used for data analysis (Figure 1).

**Table 1:** Demographic data of cases and controls


**Characteristic**	**Aborted fetus (n = 50)**	**Control group (n = 50)**	P-value*
**Range of mother age (yr)**	26-39	25-38	
Average of mother age (yr)*	32.77 ± 4.23	32.59 ± 3.89	0.942
Average of fetus age*	12.05 ± 3.2	-
Average age of infants born*	- 38.54 ± 1.16	
**Range number of abortions**	3-4	-
Average number of abortions*	3.05 ± 1.64	-
Data presented as Mean ± SD. *P-value by Student *t* test			

### Ethical consideration 

The research was performed with the approval Ethics Committee of Islamic Azad University of Dehaghan Branch, Dehaghan, Iran (Code: IR.IAU.DEHAGHAN.REC.1397.001). Written informed consent was obtained from all candidates before taking samples.

### Statistical analysis

The statistical analysis was performed using the SPSS software (Statistical Package for the Social Sciences, version 22; SPSS, Chicago, IL, USA) and Chi-square ($\chi^{2}$) test by analysis of logistic regression; p < 0.05 was considered as the level of significance and A/G distribution was in agreement with the Hardy-Weinberg equilibrium (HWE). Odds ratio (OR) and p-value with 95% confidence interval (CI) were considered to estimate the risk RSA for 820A/G variant in the *IGF-2* gene in the study group with RSA and control groups. Student's *t* test was used to compare the mean age of the mothers between the two groups and Fisher's exact test for p- value.

## 3. Results

In present study, there was no significant difference between the mean age of mothers, the mothers of the control group with age 32.59 ± 3.89 yr compared to the mothers of aborted fetuses with age 32.77 ± 4.23 (p = 0.942) (Table I). The allelic and genotypic frequency of 820 A/G in the *IGF-2* gene was studied in case and control groups. The ancestral allele A is considered as the reference. Based on the results of the statistical analysis revealed, the G allele is effective in recurrent abortions (p = 0.015). Considering the AA-genotype, as reference and analysis of other genotypes relative to this position obtained a statistically significant relationship (p = 0.04 for AG- genotype and p = 0.935 for GG-genotype). The AG genotype is effective in the incidence of recurrent miscarriage; the calculated OR showed that A/G genotype of the *IGF-2* gene at 820 locus increased the incidence of spontaneous abortion more than three-fold. Considering the phenotypic status to the advantage of allele dominance in G, a significant relationship was obtained. The statistical analysis results are presented in Table II.

**Table 2:** The distribution of genotypes and alleles of 820A/G in the IGF-2 gene in control and aborted fetus s with RSA


**Genotype/Allele**	**Control group**	**Aborted fetus **	**Odds ratio**	**CI%95**	P-value*
**AA**	25 (50)	12 (24)	_	Reference	-
**AG**	18 (36)	27 (54)	3.274	1.015-10.561	0.04
**GG**	7 (14)	11 (22)	1.048	0.342-3.210	0.935
**A**	68 (68)	51 (51)	- Reference	-
**G**	32 (32)	49 (49)	2.042	3.627-1.149	0.015
**GG+AG (G+)**	25 (50)	38 (76)	3.167	1.349-7.435	0.008
**AA+AG (A+)**	43 (86)	39 (78)	0.577	0.204-1.636	0.301
Data presented as n (%).*P-value by Fisher's exact test					

## 4. Discussion

The cause and mechanism underlying the traits and conditions that induce recurrent abortion is an important challenge for current genetics. Now, it is well-accepted that some human traits depend on the parent from whom the gene responsible for the trait is inherited. Obviously, the zygotes that were generated by maternally or paternally derived chromosomes could not survive to term. Development failure results from the entire chromosome complement inherited from only one parent. Comparison of gross morphologic complementarity of the phenotypes resulting from paternal sets of chromosomes versus maternal sets suggests that paternal genetic contribution is important for placental development, while maternal contribution is essential for proper embryo development (27).

Recently, frequent reports indications a connection between pregnancy complications such as restricted embryonic growth and RSA and the role of the *IGF-2* gene.* IGF-2* is known as a strong mitogen and is part of a cluster of imprinting genes on human chromosomes. Most imprinting genes are associated with embryo growth. The 820 A/G variation is a functional polymorphism and alter the primary sequence of encoded protein. This variation at the genomic level strongly affects the transcription level and alter *IGF-2* expression status. Thus, mRNA transcription of the *IGF-2* gene in the presence of this polymorphism will change quantatively. The G allele of the *IGF-2* gene and plasma levels of *IGF-2* protein as well as* IGF-2* mRNA shows positive correlation. Therefore, heterozygote A/G at position 820 of this gene could results in loss of genomic imprinting manner in *IGF-2* expression (25, 26, 28, 29).

The expression of the paternal allele is due to the changes in the epigenetic, including changes in the DNA structure such as DNA methylation. Imprinting genes or gene expression regulation is conducted via chromatin fiber change. The loss of the genetic imprinting (LOI) can result in a loss of the allele and imbalance (loss of function or increase in it) in a rate of the gene product and possibly also lead to the phenotypic outcome (30). Thus, the LOI leads to the loss of normal growth and development of the fetus. In order to clarify *IGF-2* gene significance in the growth and development of pre-natal, Lighten and colleagues analyzed *IGF* genes and their receptors (*IGF-1*-*IGF-2*) expression in chorionic cells. Transcripts were evident that both genes and their receptors exist in human zygote cells before implantation. Although, according to the ligands, only transcripts of the *IGF-2* ligand was detected. Based on their results, while before implantation, the parental allele function is blocked, genomic imprinting participated at the period of 8-cell embryo (31, 32). Therefore, it can be assumed that in RSA, the function of the genetic imprinting mechanism is not started, which results at the end of the growth of the organism (25).

Ostojic and co-worker have reviewed the genetic background of idiopathic RSA and the contribution of genetic changes to *IGF-2* and *H19* imprinting genes. This case-sectional study determined the relationship between *IGF-2* 820A/G and H19 *HhaI* gene variant RSA susceptibility. There was a significant difference in the frequency of *IGF-2* 820A/G in men with RSA compared to healthy men (p < 0.0001). There was no difference in the distribution of this genotypic women's groups. The presence of *IGF-2* the 820 variant among the husbands of these women in RSA couples can affect the expression of *IGF-2* in the placenta and embryo and represents a risk factor for RSA sensitivity (26). In addition, a study of 107 placental tissue samples from Ukraine reported that aborted embryos at 10-15 wk of pregnancy, carrier fetus with 820 A/G genotype, has seven times more chance to develop RSA as compared to the AA genotype (25). This study is also in line with the findings of Koukoura and colleagues from Greece who studied 31 placentas with fetal growth restriction (FGR) and found decline in IGF2 mRNA levels and LOI among the abnormal placentas. The epigenetic mechanism that regulates the genetic imprinting of the *IGF-2* gene leads to FGR and induce significantly the reduction at the level of *IGF-2* mRNA (15).

Although our study shows that the chance of RSA due to paternal G allele is increased about three times, this finding is still meaningfully lower than expected in comparison to the other study (Table III). The high rate of consanguinity marriage may introduce other possible genetic factors contributing to the development of RSA.

**Table 3:** A Comparative view of this study (Fars-Iran) with Croatia and Ukraine


**IGF-2 Genotype**		**Control **	**Patient **	**OR**	**P-value**
**A/G genotype**					
	**Iran**	18 (36), n = 50	27 (54), n = 50	3.274	0.04
	**Croatia**	28 (24.8), n = 113	54 (47.8), n = 113	3.274	< 0.0001
	**Ukraine**	9 (0.225), n = 40	74 (0.692), n = 107	7.7239	< 0.01
**G/G genotype**					
	**Iran**	7 (14), n = 50	11 (22), n = 50	1.048	0.935
	**Croatia**	22 (19.5), n = 113	44 (38.9), n = 113	- < 0.0001
	**Ukraine**	27 (0.675), n = 40	22 (0.206), n = 107	- < 0.0001
Data presented as n (%); OR and P-value by Fisher's exact test					

## 5. Conclusion

This study indicates that carriers of GG or A/G genotype are three times more likely to have recurrent abortion in our population.

##  Conflict of Interest

There is no conflict of interest in this study.
